# Predicting Social Cognitive Outcomes in Adolescent-Onset Schizophrenia: A Hierarchical Analysis of Pharmacogenetic, Clinical, and Environmental Factors

**DOI:** 10.3390/jcm15124472

**Published:** 2026-06-09

**Authors:** Bianca Oana Bucatos, Nilima Rajpal Kundnani, Marius Papurica, Nicoleta Ioana Andreescu, Liana Dehelean, Ana-Maria Romosan, Radu Ștefan Romosan, Adriana Cojocaru, Laura Alexandra Nussbaum

**Affiliations:** 1 Department of Neurosciences-Psychiatry, “Victor Babes” University of Medicine and Pharmacy, 3000417 Timisoara, Romania; bianca.bucatos@umft.ro (B.O.B.); romosan.radu@umft.ro (R.Ș.R.); 2 PhD School Department, “Victor Babes” University of Medicine and Pharmacy, 300041 Timisoara, Romania; 3 University Clinic of Internal Medicine and Ambulatory Care, Prevention and Cardiovascular Recovery, Department VI Cardiology, “Victor Babes” University of Medicine and Pharmacy, 300041 Timisoara, Romania; 4 Research Centre of Timisoara, Institute of Cardiovascular Diseases, “Victor Babes” University of Medicine and Pharmacy, 300041 Timisoara, Romania; 5 University Clinic of Anaesthesia and Intensive Care, Department X Surgery II, “Victor Babes” University of Medicine and Pharmacy, 300041 Timisoara, Romania; 6 Department of Microscopic Morphology, Discipline of Genetics, Genomic Medicine Centre, “Victor Babes” University of Medicine and Pharmacy, 2 Eftimie Murgu Square, 300041 Timisoara, Romania; andreescu.nicoleta@umft.ro; 7 Centre for Cognitive Research in Neuropsychiatric Pathology, “Victor Babes” University of Medicine and Pharmacy, 300041 Timisoara, Romania; 8 Department of Neurosciences-Pedopsychiatry, “Victor Babes” University of Medicine and Pharmacy, 300417 Timisoara, Romania

**Keywords:** schizophrenia, personalized psychiatry, adolescents, empathy, theory of mind, social cognition, pharmacogenetics, CYP2D6

## Abstract

**Background:** Socio-cognitive deficits constitute a core and persistent feature of adolescent-onset schizophrenia, significantly impairing functional outcomes. However, the interplay between genetic metabolic markers such as CYP2D6 and specific socio-cognitive phenotypes remains poorly understood. **Methods:** This cross-sectional study included 73 adolescents with schizophrenia and 58 matched healthy controls. Theory of Mind (ToM) was evaluated using the Reading the Mind in the Eyes Test (RMET), while empathy was assessed with the Cambridge Empathy Quotient. Symptom severity was measured via the Positive and Negative Syndrome Scale (PANSS). CYP2D6 polymorphisms were genotyped using RT-PCR, classifying participants as Normal or Reduced (Intermediate) metabolizers. Hierarchical multiple regression analyses were performed, controlling for sex, IQ, and psychosocial factors. **Results:** Patients demonstrated significantly lower RMET and empathy scores compared to controls. Reduced CYP2D6 metabolizers exhibited poorer ToM performance and more severe negative symptoms. The final RMET model accounted for 88.8% of variance (*p* < 0.001), with CYP2D6 status emerging as a significant independent predictor (β = 0.178, *p* = 0.005), alongside IQ and negative symptoms. In contrast, the empathy model explained 49.0% of variance, with CYP2D6 effects fully mediated by negative symptom severity. **Conclusion:** Adolescents with reduced CYP2D6 metabolic activity exhibit greater negative symptom burden and impaired social-cognitive functioning. Our findings reveal a double dissociation: ToM functions as a stable, biologically anchored trait, while empathy serves as a state-dependent construct primarily driven by the negative syndrome. These insights advocate for the integration of pharmacogenetic stratification in the treatment of early-onset schizophrenia.

## 1. Introduction

Theory of Mind (ToM) refers to the capacity to infer and attribute mental states, beliefs, intentions, emotions, and desires to oneself and others, enabling the prediction and interpretation of behavior in social contexts. This ability represents a central component of social cognition and plays a crucial role in communication, empathy, and interpersonal functioning. Developmentally, ToM begins to emerge in early childhood and continues to mature throughout adolescence, paralleling structural and functional maturation of frontal and temporal brain networks involved in social information processing [1,2,3,4]. Recent work also underscores the importance of psychometrically robust ToM measures in schizophrenia research, and a reliability-generalization meta-analysis reported acceptable reliability for the Reading the Mind in the Eyes Test (RMET) across schizophrenia samples [5,6]. Impairments in ToM have been consistently documented across psychiatric conditions, but they are particularly robust and clinically relevant in schizophrenia. Early theoretical models proposed that disturbances in mentalizing may directly contribute to hallmark symptoms such as delusions and thought disorder [7,8].

Schizophrenia is increasingly conceptualized as a disorder characterized not only by psychotic symptoms but also by profound social-cognitive dysfunction [9,10,11], a view reinforced by recent cross-regional meta-analytic and international-perspective work on social cognition in schizophrenia [12,13]. Meta-analytic evidence demonstrates moderate to large impairments in ToM across illness stages, including first-episode psychosis and remission [14,15], while clinical work has further characterized ToM impairment in relation to both symptom severity and neurocognition in schizophrenia [16]. These deficits are not fully accounted for by general intellectual decline, supporting the notion that social cognition constitutes a partially distinct neurocognitive domain [17,18].

Neuroimaging studies further support this perspective, demonstrating that mentalizing relies on a network involving the medial prefrontal cortex, superior temporal sulcus, temporo-parietal junction, anterior cingulate cortex, and inferior frontal gyrus [19,20,21,22]. Recent reviews have continued to emphasize the social-cognitive and neural mechanisms that underlie social functioning in schizophrenia [23], while newer neuroimaging work in first-episode psychosis has extended this framework to intrinsic cerebellar functional connectivity related to social cognition and ToM [24]. Structural and functional abnormalities in these regions have been repeatedly observed in schizophrenia [25]. Together, these findings suggest that ToM impairment represents a core feature of schizophrenia rather than a secondary consequence of acute psychopathology. Recent sib-pair studies further support the view of ToM deficits as a potential endophenotype, showing impairments linked to clinical liability markers (such as basic symptoms and psychotic-like experiences) even in unaffected siblings [26].

Adolescence represents a particularly relevant developmental window for studying these mechanisms. Social cognition continues to mature during this period, while early-onset schizophrenia is associated with more severe neurodevelopmental impairment and poorer long-term outcomes [27,28,29]. Recent adolescent-focused work has confirmed that neurocognitive and social-cognitive impairments are detectable in both symptomatic and remitted early-onset schizophrenia spectrum disorders, with RMET performance among the domains differentiating symptomatic from remitted youth [30]. Also, a narrative review of adolescent-onset schizophrenia similarly emphasized the developmental severity, treatment complexity, and poorer long-term outcomes of this subgroup [31]. Despite this, relatively few studies have examined ToM in adolescent populations, and even fewer have investigated biological factors that may influence social-cognitive functioning in early-onset schizophrenia.

Social cognition is among the strongest predictors of functional outcome in schizophrenia [17,32,33,34,35]. Deficits in mental state attribution are associated with impaired social integration, reduced occupational functioning, and poorer quality of life [32,36,37]. Recent work has further emphasized the role of genetic factors alongside clinical variables in explaining variability in social functioning among patients with schizophrenia [38]. Other recent systematic review evidence suggests that social cognition is linked to premorbid adjustment in psychosis [39], and contemporary meta-analytic work has further examined how emotion processing relates to symptoms and social functioning across psychotic disorders [40,41]. Social cognition explains unique variance in functional outcomes beyond traditional neurocognitive measures [36]. Consequently, social cognitive training interventions have been developed, and meta-analytic evidence supports their modest but meaningful impact on functional recovery [42,43], a conclusion reinforced by a meta-analytic work on social-cognitive training in schizophrenia, conducted by Yeo et al. in 2022 [9].

Among symptom domains, negative symptoms demonstrate the most consistent association with social-cognitive dysfunction. Avolition, anhedonia, blunted affect, and social withdrawal are strongly correlated with impaired ToM performance [44,45,46,47]. Recent evidence in children and adolescents with early-onset psychosis and clinical high risk for psychosis has further highlighted the prominence of negative symptoms early in the illness course [48], while a systematic review linked negative symptoms with ToM and other cognitive deficits in first-episode psychosis and high-risk populations [49]. Consensus frameworks such as MATRICS and CAINS validation studies emphasize the centrality of negative symptoms in long-term disability [50,51]. More recent cognitive models also suggest that dysfunctional belief systems may help maintain or exacerbate negative symptoms in schizophrenia spectrum disorders [52]. In contrast, positive symptoms show weaker and less stable associations with mentalizing ability [45].

While psychosocial adversity contributes to schizophrenia vulnerability [53], biological moderators of social cognition remain less explored. Pharmacogenetics, particularly variation in cytochrome P450 enzymes, has emerged as a clinically relevant factor influencing antipsychotic response. Literature reviews of long-term outcomes in schizophrenia spectrum disorders have renewed attention on the functional role of CYP2D6 and CYP2C19 in symptom and cognitive trajectories [54]. CYP2D6 is highly polymorphic and plays a central role in the metabolism of risperidone and aripiprazole [55,56,57]. Genetic variation results in poor, intermediate, normal, and ultrarapid metabolizer phenotypes that significantly influence plasma concentrations and adverse-effect profiles [57,58,59]. More broadly, recent work has also highlighted pharmacogenomic and genomic influences on symptom severity, cognition, and emerging clinical stratification in schizophrenia [60,61].

Second- and third-generation antipsychotics differ in anticholinergic burden, which can exacerbate cognitive and social-cognitive impairments. In our sample, antipsychotics with higher anticholinergic load (e.g., olanzapine) were considered alongside CYP2D6 metabolism, as evidence highlights their contribution to cognitive side effects in adolescents [60]. A recent systematic review of pharmacogenetic testing to guide antipsychotic treatment found that pharmacogenomics-guided prescribing (with emphasis on CYP2D6 among other genes) showed benefits or no harm for clinical outcomes (including adverse drug reactions and symptom improvement) and was potentially cost-effective, although larger randomized controlled trials with broad panels are still needed [62].

Clinical Pharmacogenetics Implementation Consortium (CPIC) guidelines provide standardized genotype-to-phenotype translation for CYP2D6 [59]. Evidence indicates that reduced metabolizers may experience increased exposure and side-effect burden, whereas ultrarapid metabolizers may demonstrate subtherapeutic response [57,58,59,60,61,63,64]. Pediatric and adolescent populations may exhibit amplified genotype-related variability due to developmental pharmacokinetics [65].

Pharmacogenetic research in Eastern European populations remains limited, although some studies have explored the clinical relevance of CYP2D6 variability and pharmacogenetic testing in psychiatric populations. Emerging local data highlight the feasibility and clinical utility of implementing personalized pharmacogenetic approaches in routine psychiatric practice [66,67,68,69]. Broader frameworks of personalized psychiatry advocate integration of pharmacogenetic testing into structured clinical decision-making and dimensional models of psychiatric symptomatology [70,71]. Similar dimensional approaches examining relationships between symptom clusters and adaptive coping strategies have been reported across major mood disorders, reinforcing the value of cluster-based psychopathological assessment in clinical practice [72].

Despite advances in pharmacogenetics and social cognition research, the relationship between CYP2D6 metabolizer status and mentalizing capacity remains largely unexplored. Given the established link between negative symptoms and ToM impairment, it is plausible that pharmacogenetic variability may indirectly influence social cognition through its impact on residual symptom burden.

### Objectives

This study aimed to assess ToM performance in adolescent patients diagnosed with schizophrenia who underwent pharmacogenetic testing for metabolic anomalies related to CYP2D6 (using an HC sample for comparison) and to investigate the multidimensional predictors of socio-cognitive performance in this sample. Specifically, the study sought to determine the relative and cumulative contributions of intelligence (IQ), CYP2D6 metabolizer status, and negative symptom severity to ToM performance (RMET scores), and to assess whether the same biological and clinical factors may predict scores in the Empathy Quotient (EQ), controlling for demographic and environmental factors (sex and psychosocial context, familial stability).

We hypothesize the following:

**H1.**  *Reduced CYP2D6 metabolizer status would be associated with poorer ToM (RMET) performance, even after controlling for IQ and negative symptoms, reflecting a direct biological influence on this trait-like domain of social cognition.*

**H2.**  *Negative symptom severity (PANSS-N) would be the strongest predictor of both RMET and EQ scores, but would fully mediate the relationship between CYP2D6 metabolizer status and empathy, consistent with empathy being more state-dependent.*

**H3.**  *The predictive model for RMET would explain substantially more variance than the model for EQ, supporting a double dissociation between these two components of social cognition.*

## 2. Materials and Methods

This cross-sectional observational case–control study was conducted at the Child and Adolescent Psychiatry Clinic in Timișoara, Romania. All procedures were approved by the institutional Ethics Committee, and written informed consent was obtained from the legal guardians of all participants prior to inclusion. The study was carried out in accordance with the Declaration of Helsinki [73].

The cross-sectional observational case–control design was selected because it efficiently captures associations between pharmacogenetic factors (CYP2D6 metabolizer status), clinical variables, and social-cognitive outcomes at a single time point during clinical stabilization. This approach is particularly suitable for adolescent-onset schizophrenia, where longitudinal follow-up is often challenging due to developmental changes, treatment variability, and attrition. It also enables direct comparison with a matched healthy control group while preserving high ecological validity through a naturalistic treatment protocol.

This design choice is supported by recent studies in adolescent and early-onset schizophrenia social cognition research. For instance, Jepsen et al. employed a cross-sectional design to examine associations between adaptive functioning, social cognition, and neurocognition in first-episode early-onset schizophrenia spectrum disorders [74]. Similarly, Gaafar et al. used an observational case–control approach to investigate social cognition and metacognition in relatives at clinical high risk for psychosis [75]. Cross-sectional studies remain prevalent (e.g., ~67% in recent reviews of social cognition in psychosis) as they provide rapid, cost-effective insights into trait-like deficits while generating hypotheses for future longitudinal work.

**Figure 1:**
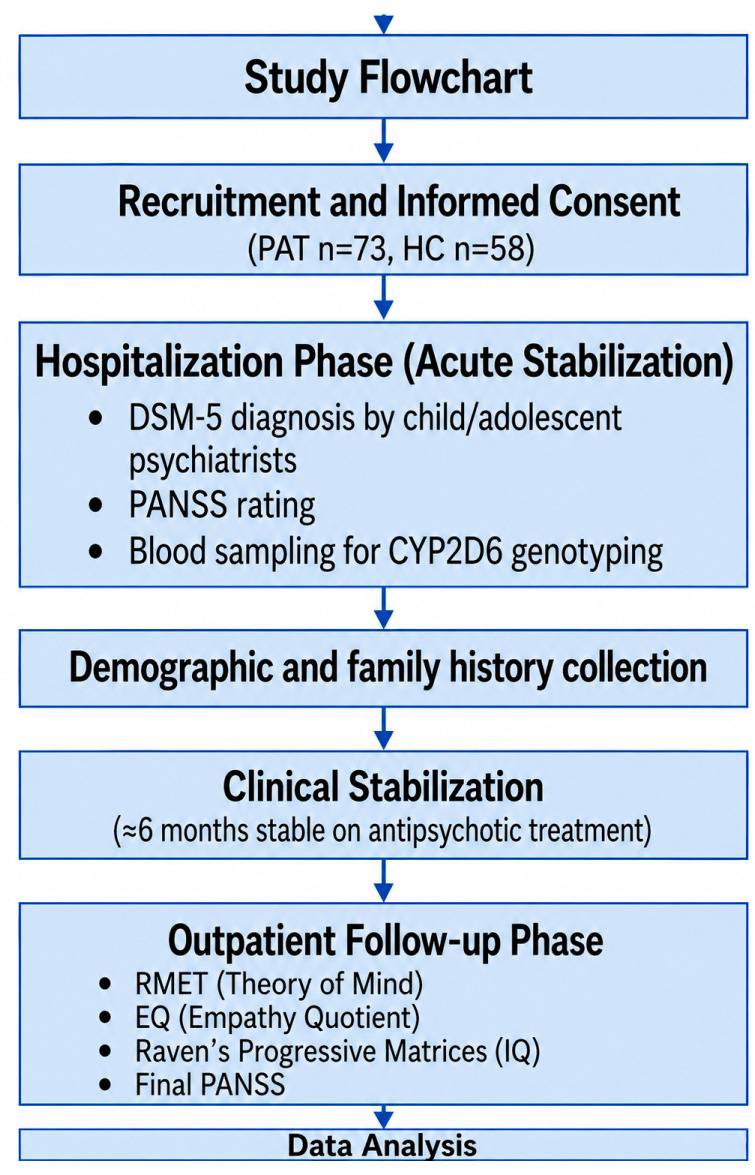
Study flowchart. PAT = patient group; HC = healthy controls.

The clinical group included 73 adolescents aged between 13 and 18 years, diagnosed with schizophrenia, according to DSM-5 diagnostic criteria [76], by their attending physician upon admission to the hospital. Exclusion criteria were refusal to participate and active substance abuse. A comparison group of 58 healthy adolescents, matched for age, sex, and intellectual level, was recruited from the community. Clinical assessments and biological sampling were performed during hospitalization. Following clinical stabilization and remission, participants completed social-cognitive assessments during routine outpatient follow-up using secure online questionnaires. Figure 1 presents the study flowchart:

Environmental and hereditary factors were assessed through clinical interviews and family history records. Psychosocial context was operationalized based on the stability of intrafamilial relationships, categorized as either “stable” (supportive, cohesive environments) or “dysfunctional” (characterized by high expressed emotion, conflict, or lack of cohesion). Family history was recorded as the presence or absence of first- or second-degree relatives with a diagnosis of a major psychiatric disorder, including schizophrenia-spectrum disorders, bipolar disorder, recurrent depression, or substance abuse disorders.

ToM was evaluated using RMET, a widely used measure of mental state attribution from facial cues [77]. Empathy was assessed using the Cambridge Empathy Quotient (EQ), a validated self-report measure of cognitive and affective empathy [78]. Both instruments have been extensively used in cross-cultural and clinical research and have been previously applied in Romanian studies investigating social cognition. Symptom severity was assessed using the Positive and Negative Syndrome Scale (PANSS), a validated instrument widely used to quantify positive, negative and general psychopathology symptoms in schizophrenia, which has been extensively applied in European and Romanian psychiatric research, including studies conducted in Romanian clinical populations [79]. Intellectual functioning was assessed using Raven’s Progressive Matrices, a non-verbal measure of general intelligence frequently used in clinical and developmental research [80].

At the time of assessment, all participants were receiving antipsychotic treatment as prescribed by their primary treating physicians. To maintain the naturalistic design of the study and ensure high ecological validity, treatment protocols, including drug choice and dosage, were not standardized or manipulated by the research team. All participants were required to be on a stable medication regimen for at least 6 months prior to social cognitive testing to ensure clinical stability.

Pharmacogenetic testing targeted three clinically relevant CYP2D6 alleles: CYP2D6*3 (rs35742686), CYP2D6*4 (rs3892097), and CYP2D6*41 (rs28371725). These alleles were chosen due to their established impact on enzyme function and relevance to the metabolism of commonly prescribed antipsychotics such as risperidone and aripiprazole. Peripheral venous blood samples (3 mL EDTA tubes) were collected following written consent. We performed DNA sampling for genotyping of single-nucleotide polymorphisms (SNPs) by real-time polymerase chain reaction (RT-PCR), with CYP* allelic variants determined by allele-specific fluorescence measurement using allelic discrimination software (QuantStudio 6, version 1.6.1, Applied Biosystems™ TaqMan^®^ Assays, Foster City, CA, USA). Genotype-to-phenotype classification followed CPIC recommendations [59]. A total of 73 participants were genotyped and initially classified into three categories based on CYP2D6 enzymatic activity: normal metabolizers (NM), intermediate metabolizers (IM), and poor metabolizers (PM). Due to the low frequency of the poor metabolizer phenotype (*N* = 2), these two patients were excluded from the study, and the analysis was carried out only using the 73 remaining IM patients.

### Statistical Analysis

Statistical analyses were performed using IBM SPSS Statistics version 20. This version continues to be employed in recent psychiatric and medical research and has proven effective for the non-parametric and regression analyses conducted in this study [81,82,83]. Data were first screened for normality, outliers, and missing values. Descriptive statistics were calculated for all demographic and clinical variables. Normality was assessed using the Kolmogorov–Smirnov and Shapiro–Wilk tests, which indicated that most variables were not normally distributed. Therefore, non-parametric tests were used for group comparisons (Mann–Whitney U and Wilcoxon signed-rank tests), which are robust alternatives to parametric tests when data are non-normal and widely used in schizophrenia studies to handle skewed clinical scores. Categorical variables were analyzed using the χ^2^ test. Associations between variables were examined using Spearman correlation coefficients, appropriate for ordinal or non-normally distributed data in clinical samples [84,85,86,87].

To identify potential covariates, bivariate correlations (Spearman’s rho) and non-parametric testing were conducted between all independent variables (age, IQ, PANSS subscales, psychosocial context, and family history) and the two primary social cognitive outcomes (RMET and EQ). Variables that did not demonstrate a significant association with the outcomes were excluded from the final regression models to ensure model parsimony.

Two separate hierarchical multiple regression models were constructed to identify the predictors of ToM (RMET) and empathy (EQ). For both models, predictors were entered in four successive blocks: block 1: intelligence (IQ) and sex were entered as baseline control variables; block 2: CYP2D6 metabolizer status was introduced to assess the biological contribution (coded as 0 = normal, 1 = intermediate); block 3: PANSS-N (Negative symptoms) was added to determine the incremental variance explained by clinical symptomatology; block 4: psychosocial context was entered to account for environmental influences (coded as 0 = stable environment, 1 = dysfunctional families). The health of each model was assessed using standard diagnostics. This hierarchical method, used to evaluate the relative contributions of predictors while controlling for confounders, allows the sequential entry of theoretically relevant blocks and has been effectively applied in recent psychiatric research to disentangle biological, clinical, and environmental influences on outcomes in schizophrenia [88,89]. Multicollinearity was evaluated using Variance Inflation Factors (VIFs) and Tolerance values; VIF values < 3.0 were considered acceptable. Independence of errors was verified using the Durbin-Watson statistic, with values between 1.5 and 2.5 considered optimal. Residual plots were examined to confirm homoscedasticity and the normality of residuals. Standardized (β) and unstandardized (B) coefficients were reported for each model to facilitate comparison. The significance level for all tests was set at *p* = 0.05.

## 3. Results

The results of the Kolmogorov–Smirnov and Shapiro–Wilk normality tests can be observed in Table 1.

### 3.1. Comparison PAT vs. HC

The study included two samples matched for sex and age: PAT and HC. Demographic and clinical characteristics for the PAT and HC samples are presented in Table 2.

PAT presented significantly reduced RMET and total EQ scores compared to HC. There were no significant differences in IQ scores between the two samples, as shown in Table 3.

### 3.2. Preliminary Analysis of the PAT Sample

The mean age of the PAT sample was 15.16 (SD = 1.19) years, ranging from 14 to 18 years. Age did not correlate significantly with RMET, EQ, IQ and PANSS (P, N, G, and total) scores (*p* > 0.05).

The differences between scale scores (RMET, total EQ, PANSS total and sub-scores, and IQ), taking into account the demographic and clinical parameters, are presented in Table 4. Female patients with SCZ had significantly higher RMET and IQ scores and significantly lower PANSS scores (N, G, and total scores). Patients with IM scored significantly lower in RMET and EQ, whilst having significantly higher PANSS (N, G and total) and IQ scores than patients with normal metabolism. Only total EQ scores were influenced by the choice of antipsychotic treatment (minimal/low impact on CYP2D6 vs. moderate/high impact on CYP2D6); patients receiving antipsychotics with minimal/low impact on CYP2D6 presented with significantly higher total EQ scores. Patients with a family history of psychiatric conditions scored significantly lower on RMET scores, while simultaneously exhibiting significantly higher PANSS scores (N, G and total scores). Patients originating from disorganized families also presented with significantly lower RMET and EQ scores, and significantly higher PANSS scores (P, N, G, and total scores).

Preliminary bivariate analyses using Spearman’s rho (Table 5) revealed that social cognitive measurements (RMET and EQ scores) were strongly associated with specific clinical dimensions. Negative symptom severity (PANSS-N) demonstrated a near-perfect inverse correlation with RMET (rs = −0.945, *p* < 0.0001) and a robust inverse correlation with EQ (rs = 0.681, *p* < 0.0001). By contrast, positive symptoms (PANSS-P) did not reach statistical significance in their association with either RMET (*p* = 0.13), or EQ (*p* = 0.16).

While General Psychopathology scores (PANSS-G) were significantly correlated with social outcomes, their high collinearity with PANSS-N (rs = 0.871, *p* < 0.0001) suggested redundant predictive value. Consequently, to ensure model parsimony and focus on the most theoretically relevant clinical driver (negative symptoms have been consistently identified in the literature as the strongest clinical correlate of social-cognitive impairment in schizophrenia), only PANSS-N scores were selected for inclusion in the subsequent hierarchical regression models.

### 3.3. Differential Predictors of Social Cognition

Predictor variables for the hierarchical regression models were selected based on a two-step process. First, variables were included based on their theoretical relevance to social cognition in schizophrenia (IQ, sex, and psychosocial context). Second, clinical symptoms were screened using bivariate correlation; although all PANSS subscales were measured, only the Negative Symptom subscale (PANSS-N) was included in the final models due to its robust and significant association with both RMET and EQ scores, whereas positive and general symptoms showed weak or non-significant relationships. Preliminary analyses showed that age and family history of psychiatric disorders did not significantly correlate with the primary outcomes (RMET and EQ); therefore, to maintain model parsimony, they were not included as predictors in the final hierarchical regression models. The specific type and dosage of antipsychotic medication were not controlled for, as treatment was left to the discretion of the patients’ primary physicians, and several patients were receiving a combination of antipsychotics at the time of the assessment.

A four-step hierarchical entry method was employed to determine the unique variance contributed by each factor:

- Step 1: Intelligence (IQ scores) and Sex were entered as baseline control variables.
- Step 2: CYP2D6 metabolizer status was introduced to assess the primary biological contribution.
- Step 3: PANSS-N scores were added to evaluate the incremental predictive value of clinical symptomatology.
- Step 4: Psychosocial context was included to account for environmental influences.

To maintain a conservative and rigorous analysis, demographic (sex) and environmental (psychosocial context) factors were retained in the models regardless of their individual significance. This approach ensured that the observed effects of genetic and clinical predictors were adjusted for potential confounding variables.

#### 3.3.1. Hierarchical Prediction of ToM—RMET Scores

To evaluate the incremental predictive value of demographic, genetic, and clinical factors on social cognition, a 4-step hierarchical ordinary least squares (OLS) regression analysis was conducted with RMET total scores as the dependent variable.

A hierarchical regression was conducted to predict RMET scores. In Step 1, intelligence and sex accounted for 49% of the variance, though only intelligence was a significant predictor (β = 0.525, *p* < 0.0001). The addition of CYP2D6 metabolizer status in Step 2 significantly increased the explained variance by 22.5% (R^2^ = 0.715, Adjusted R^2^ = 0.703, *p* < 0.0001), identifying it as a major biological determinant, metabolizer status being significantly and negatively associated with RMET scores (B = −9.374, β = −0.548, t = −7.38, *p* < 0.0001). Step 3 (PANSS-N) added a further 17.6% (ΔR^2^ = 0.176, *p* < 0.0001). The full model now accounted for 89.1% of the total variance (R^2^= 0.891, Adjusted R^2^ = 0.885; F(4, 68) = 139.44, *p* < 0.0001). In this step, PANSS (N) emerged as the strongest standardized predictor in the model (B = −2.295, β = −0.723, t = −10.51, *p* < 0.0001), while the standalone effect of metabolizer status attenuated, but remained significant (B = −2.760, β = −0.161, t = −2.73, *p* = 0.008). The addition of psychosocial context to the final model did not significantly improve it (ΔR^2^ = 0.000, F(1, 67) = 0.204, *p* = 0.653). The final model explained 89.2% of total variance (R^2^ = 0.892, Adjusted R^2^ = 0.884; F(5, 67) = 110.29, *p* < 0.0001). In this final full model, psychosocial context was not significantly associated with RMET scores (B = −0.372, β = −0.020, *p* = 0.653). As can be seen in Table 6, in the final model, PANSS-N emerged as the strongest predictor (β = −0.70), followed by metabolizer status (β = −0.18) and IQ (β = 0.16). Psychosocial context and sex did not significantly contribute to the model (*p* > 0.05).

#### 3.3.2. Hierarchical Prediction of Empathy—EQ Scores

Another hierarchical regression model (Table 7) was conducted to identify factors influencing empathy (EQ scores) in remitted adolescent patients with schizophrenia. The hierarchical regression for EQ scores (Table 7) revealed a distinct predictive pathway compared to RMET. In Step 1, intelligence (IQ) and sex explained 26.9% of the variance (R^2^ = 0.269, Adjusted R^2^ = 0.248). At this stage, IQ was a highly significant positive predictor (B = 0.350, β = 0.497, t = 4.56, *p* < 0.0001), while sex was not significant (*p* = 0.614). The inclusion of metabolizer status during step 2 explained an additional 4.6% of the variance, representing a statistically significant improvement (ΔR^2^ = 0.046, F(1, 69) = 4.68, *p* = 0.034). Total variance explained rose to 31.6% (R^2^ = 0.316, Adjusted R^2^ = 0.286). In this step, metabolizer status was significantly and negatively associated with empathy scores (B = −3.931, β = −0.249, t = −2.16, *p* = 0.034). In step 3, PANSS (N)—Negative symptoms, was entered. This step accounted for a major 15.9% increase in explained variance, which was highly significant (ΔR^2^ = 0.159, F(1, 68) = 20.62, *p* < 0.0001). The model now accounted for 47.5% of total variance (R^2^ = 0.475, Adjusted R^2^ = 0.444; F(4, 68) = 15.37, *p* < 0.0001). Moreover, upon entering this model, PANSS (N) emerged as a powerful negative predictor (B = −2.013, β = −0.687, t = −4.54, *p* < 0.0001), while the previously significant effects of both IQ (*p* = 0.223) and metabolizer status (*p* = 0.365) were rendered non-significant, indicating that their shared variance with empathy is fundamentally accounted for by the presence of negative symptoms. In the final step (Model 4), psychosocial context was entered. The addition of this variable did not alter the model’s explanatory power (ΔR^2^ = 0.000, F(1, 67) = 0.025, *p* = 0.874). The final model explained 47.5% of total variance (R^2^ = 0.475, Adjusted R^2^ = 0.436; F(5, 67) = 12.13, *p* < 0.0001). In this final full model, PANSS (N) remained the lone significant predictor of EQ scores. These results suggest that the influence of genetic metabolism on affective empathy is mediated through the severity of the negative syndrome.

## 4. Discussion

The present study aimed to elucidate the predictive architecture of social cognition in adolescent-onset schizophrenia by integrating pharmacogenetic, clinical, and environmental factors. Our findings reveal a striking divergence between the two primary domains of social cognition: ToM (assessed via RMET) appears to be a stable, biologically driven trait, whereas empathy (assessed via EQ) functions as a state-dependent construct primarily governed by the severity of the negative syndrome. We characterize these findings as divergent because they demonstrate that social cognition in adolescent schizophrenia is not a monolithic construct. Rather, it consists of distinct neurobiological pathways: a trait-like pathway for ToM that is constrained by genetic and cognitive factors, and a state-like pathway for affective empathy that is acutely sensitive to the patient’s current negative symptom burden. This pattern is consistent with evidence of dissociable cognitive and affective components of social cognition in schizophrenia and related conditions [90,91], as well as with current international syntheses arguing that social cognition should be treated as a multidimensional target in schizophrenia research and intervention [13].

Consistent with prior meta-analytic literature, adolescents with schizophrenia demonstrated significant impairments in mentalizing and empathy compared with healthy controls [14,15,32]. These deficits persisted despite comparable intellectual functioning between patients and controls, reinforcing the view that social cognition constitutes a partially distinct domain rather than a mere reflection of generalized cognitive decline [17,18]. This distinction is clinically meaningful, as ToM impairment has been shown to contribute independently to functional outcome [32,36]. Our results are also congruent with more recent work in early-onset schizophrenia spectrum disorders showing that symptomatic youth display poorer RMET and executive-function/processing-speed performance than remitted youth [30]. Adolescence represents a particularly relevant developmental window for studying these mechanisms. Social cognition continues to mature during this period, while early-onset schizophrenia is associated with more severe neurodevelopmental impairment and poorer long-term outcomes [27]. Demonstrating these deficits in an adolescent population strengthens the neurodevelopmental perspective of schizophrenia. Social-cognitive impairment appearing early in the course of illness suggests that these difficulties are not merely a consequence of chronicity, long-term medication exposure or prolonged social isolation. Instead, they could likely represent an early-emerging and clinically meaningful dimension of the disorder that may influence long-term functional trajectories. This interpretation is further supported by recent reviews emphasizing the severity and treatment complexity of adolescent-onset schizophrenia [31] and by systematic review data linking social cognition with premorbid adjustment in psychosis [39].

The hierarchical regression for RMET demonstrated exceptionally high explanatory power (R^2^ = 0.892), indicating that ToM in this population is a highly determined construct. Even after controlling for intelligence (IQ scores), CYP2D6 metabolizer status remained a robust independent predictor. The persistent significance of the genetic variable suggests that CYP2D6-related metabolic abnormalities may impose a biological ceiling on the ability to decode social cues. This supports emerging evidence for an endogenous role of CYP2D6 in the central nervous system. Beyond its well-established function in antipsychotic metabolism, CYP2D6 is expressed in key brain regions such as the thalamus and hippocampus, where it may influence the biotransformation of neuroactive amines (e.g., dopamine, serotonin) that are relevant to social-perceptual processing [20,92,93]. Recent reviews of the social-cognitive neural architecture of schizophrenia and newer imaging studies in first-episode psychosis similarly support a distributed network model that includes cerebellar contributions to ToM and social cognition [23,24]. Intelligence also remained a significant predictor, consistent with the cognitive demands of the RMET task, which requires integration of visual cues and linguistic labeling [17]. Importantly, recent reliability-generalization work supports continued use of RMET-like ToM measures in schizophrenia research, reporting acceptable reliability for RMET in schizophrenia samples [5].

By contrast, the EQ model (R^2^ = 0.475) revealed a “washout” effect of biology in favor of clinical symptomatology. While reduced metabolizer status was initially significant, its influence vanished when negative symptoms (PANSS-N) were entered into the model. This statistical pattern indicates that the genetic burden does not directly impair empathy; rather, it likely contributes to a more severe negative syndrome, which in turn acts as a barrier to empathetic resonance. Notably, intelligence was not a significant predictor of empathy (*p* = 0.224) in this study. This suggests that, unlike ToM, which relies on cognitive abilities, empathy is an affective process that is independent of IQ but highly vulnerable to the affective flattening and social withdrawal characteristic of the negative syndrome [36,44,94]. This interpretation is consistent with recent meta-analytic work showing that negative and related symptom domains help mediate links between emotion processing and social functioning across psychotic disorders [40].

One of the most important findings of the present study is the central role of negative symptoms in explaining social-cognitive deficits. Negative symptom severity showed a strong association with both RMET and EQ performance. In the empathy model, negative symptoms fully mediated the relationship with CYP2D6 metabolizer status. Avolition, anhedonia, blunted affect, and social withdrawal are strongly correlated with impaired ToM performance [44,45,46]. Recent literature further strengthens this interpretation: a meta-analysis in youth with early-onset psychosis and clinical high risk underscores the prominence of negative symptoms early in illness [48], and a systematic review in first-episode/high-risk samples links negative symptoms with deficits in ToM and executive functioning [49]. Consensus frameworks such as MATRICS and CAINS validation studies emphasize the centrality of negative symptoms in long-term disability [50,51], while contemporary cognitive models suggest that dysfunctional beliefs may contribute to the persistence of negative symptoms [52], potentially compounding social disengagement and limiting opportunities for social learning. Diminished motivation and reduced anticipatory pleasure may limit engagement in social contexts, thereby restricting opportunities for social learning and refinement of mental state attribution. Social withdrawal may additionally reduce corrective feedback, reinforcing maladaptive interpretative patterns. Thus, negative symptoms emerged as the most powerful clinical correlate of social-cognitive performance, with 94% of the total effect of residual psychopathology on ToM abilities mediated by negative symptoms in related analyses.

The pharmacogenetic findings in this study showed that reduced CYP2D6 metabolizer status was associated with poorer ToM performance and greater negative symptom severity. CYP2D6 polymorphisms significantly influence risperidone and aripiprazole plasma concentrations [57,64]. Notably, among the antipsychotics used in our sample (aripiprazole, olanzapine, risperidone, and quetiapine), those with higher anticholinergic burden and stronger D2 receptor blockade (particularly risperidone and aripiprazole) have been associated in the literature with worsening or persistence of negative symptoms such as emotional blunting and avolition [60]. This may partially explain the observed relationship between reduced CYP2D6 metabolizer status and higher negative symptom severity in our cohort, as these medications are primarily metabolized by CYP2D6. Elevated exposure among reduced metabolizers may increase susceptibility to dose-related adverse effects such as emotional blunting, sedation, or psychomotor slowing, clinical phenomena that overlap with negative symptom expression. Conversely, genotype-related variability may influence residual symptom burden through altered treatment response [55,58]. While cross-sectional design limits causal inference, the hierarchical regressions suggest that negative symptoms may serve as an intermediary between metabolizer status and affective empathy, while ToM retains a direct genetic contribution. This interpretation is consistent with the broader pharmacogenetic literature demonstrating that CYP2D6 variation influences clinical outcomes and adverse-effect burden [59,63,65]. More recent work has extended this framework by emphasizing the long-term functional relevance of CYP2D6/CYP2C19 variation [54], broader pharmacogenomic effects on symptom severity and cognitive ability [60], and the emerging clinical implications of schizophrenia genomics for precision psychiatry [61]. Our findings extend pharmacogenetic research beyond symptom reduction to include higher-order interpersonal functioning.

Across both models, sex and psychosocial context failed to reach statistical significance. While females generally show advantages in social cognition in the general population, typically outperforming males on both RMET and EQ [95,96], our data suggests that the pathological burden of adolescent schizophrenia may neutralize these natural differences. Similarly, the lack of environmental influence (stable vs. dysfunctional families) suggests that at this stage of the illness, internal neurobiological and clinical factors outweigh external social supports in determining social cognitive performance [53].

The “double dissociation” identified in this study has important clinical implications. For patients struggling with ToM deficits, which appear more trait-like and biologically anchored, traditional symptom management may not be enough. Targeted interventions such as Social Cognition and Interaction Training (SCIT) may be particularly beneficial, as meta-analytic evidence supports their efficacy in improving mentalizing and broader functional outcomes [39,50,97], and a more recent meta-analysis also supports the beneficial effects of social-cognitive training in schizophrenia [9]. However, for patients with empathy deficits, the primary therapeutic target should be reduction in negative symptoms through optimized pharmacotherapy and psychosocial interventions, as successful alleviation of apathy and withdrawal can unmask latent empathetic capacity [44]. In adolescent populations specifically, recent reviews likewise support combining optimized pharmacotherapy with psychosocial interventions to improve adherence and functional recovery [31]. Our results suggest that empathetic abilities are not lost at the genetic level, but rather overshadowed by the clinical negative symptomatology. Successful pharmacological or behavioral reduction in apathy and withdrawal may readjust a patient’s latent empathetic capacity, which is a vital component of long-term social reintegration.

### 4.1. Study Limitations

Despite the robust predictive power of our hierarchical models, several limitations must be acknowledged. While a sample of *N* = 73 was sufficient to detect the large effect sizes observed in our social cognitive models, it may be underpowered to identify subtle psychosocial or environmental influences. This limitation remains common in pharmacogenetic and social cognition research due to recruitment challenges in adolescent populations [60]. Furthermore, our cohort consisted exclusively of adolescents with schizophrenia. While this allowed for a focused analysis of early-onset pathology, the findings may not be fully generalizable to adult populations or those with late-onset schizophrenia, where neurodevelopmental trajectories and social experiences differ.

The study utilized a cross-sectional design, which limits our ability to establish definitive causal relationships. While our hierarchical regression suggests a mediating role of negative symptoms on empathy, longitudinal data would be required to track how fluctuations in symptom severity over time correlate with changes in social cognitive performance. This continues to be a widespread challenge in the field [74,75]. Future longitudinal studies with repeated assessments or structural equation modeling (SEM) are recommended to clarify temporal dynamics and confirm mediation. Although the hierarchical regression demonstrated that the predictive value of the CYP2D6 metabolizer status was attenuated upon the inclusion of clinical symptoms in the empathy model, this “statistical mediation” was not confirmed via formal SEM or bootstrapping techniques. Future research with larger datasets should employ these methods to confirm the specific paths of influence between genetics, symptoms, and social cognition, as demonstrated in recent pharmacogenomic studies using structural approaches [60].

First, the specific type and dosage of antipsychotic medication were not controlled for, as treatment was left to the discretion of the patients’ primary physicians. While this reflects real-world clinical practice, different antipsychotic profiles could theoretically influence social cognitive performance. However, our inclusion of CYP2D6 metabolizer status as a predictor partially accounts for the biological variance in how these medications are processed, which may be more relevant to neurocognitive outcomes than the nominal dosage alone. This approach aligns with recent naturalistic studies that similarly highlight the interplay between pharmacogenomics and medication variability in real-world schizophrenia samples, while underscoring the inherent limitations of not standardizing treatment [60]. Future studies should incorporate therapeutic drug monitoring to better disentangle drug-genotype interactions, as supported by recent naturalistic TDM studies in adolescent populations treated with aripiprazole and risperidone [98,99].

First, the specific type and dosage of antipsychotic medication were not controlled for, as treatment was left to the discretion of the patients’ primary physicians. While this reflects real-world clinical practice, different antipsychotic profiles could theoretically influence social cognitive performance. However, our inclusion of CYP2D6 metabolizer status as a predictor partially accounts for the biological variance in how these medications are processed, which may be more relevant to neurocognitive outcomes than the nominal dosage alone. This limitation remains relevant given the prevalence of naturalistic designs [60]. Future studies should incorporate therapeutic drug monitoring to better disentangle drug-genotype interactions [62].

The grouping of intermediate and poor metabolizers into a single “reduced metabolizer” category was a necessary statistical adjustment due to the rarity of the PM phenotype, but it may have masked subtle differences between these two genetic subgroups. This approach is common in pharmacogenetic studies of CYP2D6 [54]. Larger multi-site studies or polygenic risk scores could address this limitation in future work.

The use of CYP2D6 metabolizer status as a categorical predictor, while clinically relevant, may oversimplify the complex polygenic landscape of schizophrenia. Other genetic variants or epigenetic factors not captured in this study may also contribute to the variance in RMET and EQ scores.

While we controlled for intelligence and sex, other potential confounders, such as the duration of untreated psychosis (DUP) or specific types of antipsychotic medication (beyond simple dosage), were not included in the final regression blocks. Future studies should aim to integrate these clinical variables to further refine the predictive models. Finally, the naturalistic design regarding antipsychotic treatment represents another limitation, because although leaving medication to the primary doctor’s discretion increases the ecological validity of our findings, it also limits us from ruling out specific drug-genotype interactions.

### 4.2. Strengths and Novelty

This study has several strengths. It focuses on an understudied adolescent population, integrates pharmacogenetics with social cognition, and employs hierarchical regression analyses to explore the relative contributions of biological, clinical, and environmental factors to ToM impairment. To our knowledge, few studies have examined the relationship between CYP2D6 metabolizer status and social cognition in this age group using hierarchical regression modeling [54,60]. The results support a model in which pharmacogenetic variability influences negative symptoms, which in turn affect social-cognitive functioning, while ToM retains additional direct genetic and cognitive determinants. This integrative approach contributes to the emerging field of personalized psychiatry.

The naturalistic treatment protocol and inclusion of a matched healthy control group enhance ecological validity and generalizability to clinical practice. The hierarchical regression approach allowed clear delineation of the relative contributions of biological (CYP2D6), cognitive (IQ), clinical (negative symptoms), and environmental factors, revealing a compelling double dissociation between trait-like ToM and more state-dependent empathy. This pattern supports emerging calls for treating social cognition as a multidimensional target in schizophrenia research.

By demonstrating that negative symptoms fully mediate the effect of reduced CYP2D6 metabolism on empathy while ToM retains independent genetic and cognitive predictors, the study advances the integration of pharmacogenetics into personalized psychiatry frameworks for adolescent-onset schizophrenia. These findings have direct clinical implications for tailoring interventions, targeting negative symptoms for empathy deficits and combining pharmacogenetic-informed prescribing with social cognitive training for ToM impairments, aligning with recent evidence linking genetic and clinical factors to social functioning outcomes in schizophrenia [38].

## 5. Conclusions

The present study provides a comprehensive mapping of the factors predicting social cognitive performance in adolescents with schizophrenia.

Our results largely corroborate the hypotheses presented in the Introduction. Reduced CYP2D6 metabolizer status remained a significant independent predictor of RMET performance (β = −0.548, t = −7.38, *p* < 0.0001) alongside IQ and negative symptoms, while negative symptom severity fully mediated its effect on EQ scores. The hierarchical models confirmed the predicted double dissociation, with RMET variance reaching 89.2% compared to 47.5%for EQ.

Our results demonstrate a predictive dissociation between the cognitive and affective dimensions of social cognition. ToM appears to be a stable, neurobiologically anchored trait, primarily driven by the interplay between CYP2D6 genetic variation, general intelligence, and negative symptoms. By contrast, affective empathy (EQ) seems to be a state-dependent construct, where the influence of underlying biology is mediated by the severity of the negative syndrome. These findings may carry significant clinical implications for early-onset schizophrenia. The lack of influence from sex and psychosocial context suggests that the pathological burden of the illness overrides typical demographic and environmental buffers. This study suggests that empathy deficits are mainly linked to negative symptoms in adolescents with schizophrenia. Conversely, ToM deficits appear more foundational and may require targeted cognitive remediation strategies to bypass biological and intellectual constraints.

Ultimately, integrating pharmacogenetic markers like CYP2D6 into the clinical assessment of adolescent schizophrenia may allow for better stratification of patients. By identifying those at higher biological risk for social cognitive failure, clinicians can implement more personalized interventions during this critical developmental window to improve long-term functional outcomes and social reintegration.

## Figures and Tables

**Table 1:** Results of normality tests for data distribution.

Tests of Normality						
	Kolmogorov–Smirnov ^a^			Shapiro–Wilk		
	Statistic	df	*p*	Statistic	df	*p*
Age	0.237	56	<0.0001 **	0.827	56	<0.0001 **
RMET	0.242	56	<0.0001 **	0.792	56	<0.0001 **
EQ TOTAL	0.088	56	0.200	0.978	56	0.382
IQ-PAT	0.111	56	0.082	0.957	56	0.044 *
PANSS-P	0.344	56	<0.0001 **	0.726	56	<0.0001 **
PANSS-N	0.182	56	<00001 **	0.882	56	<0.0001 **
PANSS-G	0.204	56	<0.0001 **	0.868	56	<0.0001 **
PANSS total	0.188	56	<0.0001 **	0.892	56	<0.0001 **
Age HC	0.204	56	<0.0001 **	0.896	56	<0.0001 **
RMET-HC	0.084	56	0.200	0.973	56	0.246
EQ TOTAL HC	0.128	56	0.022 *	0.935	56	0.005 *
IQ-HC	0.100	56	0.200	0.967	56	0.135

* This is a lower bound of the true significance. ^a^ Lilliefors Significance Correction. RMET—Reading the Mind in the Eyes; EQ—Empathy Quotient; PANSS—Positive and Negative Symptom Scale; PANSS-P—Positive and Negative Symptom Scale–Positive symptoms; PANSS-N—Positive and Negative Symptom Scale–Negative symptoms; PANSS-G—Positive and Negative Symptom Scale–General symptoms; IQ—Intelligence quotient; PAT—patient sample; HC—healthy controls. * *p* < 0.05, ** *p* < 0.001.

**Table 2:** Demographic and clinical characteristics PAT vs. HC.

Sample Characteristics	PAT		HC		Chi Square Test (χ^2^) or Mann–Whitney U Test (PAT vs. HC)	*p* Value
	N = 73	%	N = 58	%		
Sex						
males	39	53.4	30	51.7	χ^2^ = 0.037	0.86
females	34	46.6	28	48.3		
CYP2D6 metabolizer status						
normal	39	53.4	-	-	-	-
intermediate	34	46.6	-	-		
Antipsychotic treatment						
aripiprazole	25	34.2	-	-	-	-
olanzapine	23	31.5	-	-		
risperidone	21	28.8	-	-		
quetiapine	4	5.5	-	-		
Antipsychotic: impact on CYP2D6						
minimal/low impact (olanzapine, quetiapine)	27	37	-	-	-	-
moderate/high impact (risperidone, aripiprazole)	46	63	-	-	-	-
Family history of psychiatric conditions						
no	48	65.8	-	-	-	-
yes	25	34.2	-	-	-	-
Psychosocial context						
Adequate/satisfactory family relationships	51	69.9	-	-	-	-
Dysfunctional families	22	30.1	-	-	-	-
Age (years): M (SD)	15.16 (1.19)		15.53 (1.24)		U = 1754	0.081

PAT—patient sample; HC—healthy controls.

**Table 3:** Mean scale scores (comparatively for PAT and HC) and the results of the Wilcoxon signed-rank test.

		M	SD	Wilcoxon Signed-Rank Test	*p*
Pair 1	RMET PAT	22.06	8.58	Z = −3.61	<0.0001 **
	RMET HC	27.98	4.14		
Pair 2	EQ-TOTAL PAT	26.52	7.92	Z = −6.47	<0.0001 **
	EQ-TOTAL HC	46.65	6.68		
Pair 3	IQ PAT	99.28	11.24	Z = −0.24	0.81
	IQ-HC	99.84	10.19		

M—mean; SD—standard deviation; RMET—Reading the Mind in the Eyes; EQ—Empathy Quotient; IQ—Intelligence quotient; PAT—patient sample; HC—healthy controls. ** *p* < 0.001.

**Table 4:** Psychiatric assessment of the PAT sample considering demographic and clinical data.

	RMET	EQ-Total	PANSS-P	PANSS-N	PANSS-G	PANSS Total	IQ Score	Age (Years)
	M (SD)	M (SD)	M (SD)	M (SD)	M (SD)	M (SD)	M (SD)	M (SD)
Sex								
males	19.89 (SD = 7.94)	24.84 (SD = 7.58)	7.48 (SD = 0.72)	11.25 (SD = 2.46)	20.3 (SD = 3.11)	40.48 (SD = 6.97)	95.66 (SD = 10.61)	15.23 (SD = 1.24)
females	24.55 (SD = 8.73)	28.44 (SD = 7.98)	7.61 (SD = 0.81)	9.32 (SD = 2.62)	18.73 (SD = 3.06)	36.11 (SD = 6.39)	103.44 (SD = 10.62)	15.08 (SD = 1.13)
Mann–Whitney U test (U, *p* value)	U = 341.5, *p* < 0.0001 **	U = 462.5, *p* = 0.03 *	U = 614, *p* = 0.53	U = 347, *p* < 0.0001 **	U = 412, *p* = 0.005 *	U = 372, *p* = 0.001 *	U = 362.5, *p* = 0.001 *	U = 625.5, *p* = 0.66
CYP2D6 metabolizer status								
normal	28.07 (SD = 2.69)	29.74 (SD = 5.11)	7.43 (SD = 0.71)	8.56 (SD = 1.27)	17.69 (SD = 1.39)	33.87 (SD = 2.94)	104.46 (SD = 7.42)	15.1 (SD = 1.14)
intermediate	15.17 (SD = 7.79)	22.82 (SD = 8.97)	7.67 (SD = 0.81)	12.41 (SD = 2.44)	21.74 (SD = 3.27)	43.71 (SD = 6.64)	93.35 (SD = 12.02)	15.23 (SD = 1.26)
Mann–Whitney U test (U, *p* value)	U = 79.5, *p* < 0.0001 **	U = 299.5, *p* < 0.0001 **	U = 553, *p* = 0.16	U = 130, *p* < 0.0001 **	U = 169, *p* < 0.0001 **	U = 120.5, *p* < 0.0001 **	U = 292, *p* < 0.0001 **	U = 629.5, *p* = 0.69
Antipsychotic: impact on CYP2D6								
minimal/low impact (olanzapine, quetiapine)	25.41 (SD = 4.39)	31.07 (SD = 6.85)	7.48 (SD = 0.75)	9.52 (SD = 2.06)	18.67 (SD = 1.98)	36.37 (SD = 4.75)	101.81 (SD = 10.37)	15.52 (SD = 1.37)
moderate/high impact (risperidone, aripiprazole)	20.11 (SD = 9.81)	23.85 (SD = 7.32)	7.59 (SD = 0.78)	10.84 (SD = 2.93)	20.11 (SD = 3.61)	39.68 (SD = 7.85)	97.80 (SD = 11.58)	14.96 (SD = 1.03)
Mann–Whitney U test (U, *p* value)	U = 504, *p* = 0.18	U = 322, *p* = 0.001 *	U = 573.5, *p* = 0.53	U = 462.5, *p* = 0.07	U = 517, *p* = 0.23	U = 499, *p* = 0.16	U = 510.5, *p* = 0.21	U = 482, *p* = 0.09
Family history of psychiatric conditions								
no	23.83 (SD = 7.69)	27.02 (SD = 6.85)	7.43 (SD = 0.68)	9.87 (SD = 2.48)	18.87 (SD = 2.56)	36.77 (SD = 5.68)	101.18 (SD = 9.54)	15.18 (SD = 1.19)
yes	18.68 (SD = 9.33)	25.56 (SD = 9.74)	7.76 (SD = 0.87)	11.28 (SD = 2.92)	20.92 (SD = 3.96)	41.68 (SD = 8.23)	95.64 (SD = 13.41)	15.12 (SD = 1.20)
Mann–Whitney U test (U, *p* value)	U = 376.5, *p* = 0.009 *	U = 513.5, *p* = 0.31	U = 486, *p* = 0.13	U = 430, *p* = 0.04 *	U = 407, *p* = 0.02 *	U = 379.5, *p* = 0.01 *	U = 443, *p* = 0.68	U = 579.5, *p* = 0.80
Psychosocial context								
adequate/satisfactory family relationships	24.87 (SD = 7.56)	27.68 (SD = 7.31)	7.41 (SD = 0.66)	9.74 (SD = 2.47)	18.76 (SD = 2.51)	36.49 (SD = 5.61)	100.82 (SD = 9.83)	15.15 (SD = 1.17)
dysfunctional families	17.04 (SD = 9.08)	23.37 (SD = 8.61)	7.91 (SD = 9.88)	12.04 (SD = 2.91)	21.70 (SD = 4.0)	43.75 (SD = 8.61)	95 (SD = 13.53)	15.20 (SD = 1.21)
Mann–Whitney U test (U, *p* value)	U = 297.5, *p* = 0.001 *	U = 381.5, *p* = 0.03 *	U = 405, *p* = 0.03 *	U = 320.5, *p* = 0.003 *	U = 309, *p* = 0.002 *	U = 275, *p* = 0.001 **	U = 437.5, *p* = 0.14	U = 559.5, *p* = 0.98

M—mean; SD—standard deviation; RMET—Reading the Mind in the Eyes; EQ—Empathy Quotient; PANSS—Positive and Negative Symptom Scale; PANSS-P—Positive and Negative Symptom Scale—Positive symptoms; PANSS-N—Positive and Negative Symptom Scale—Negative symptoms; PANSS-G—Positive and Negative Symptom Scale—General symptoms; IQ—Intelligence quotient. * *p* < 0.05, ** *p* < 0.001.

**Table 5:** Spearman’s Rho Correlations for clinical and socio-cognitive variables (N = 73).

Variable	RMET Total	EQ Total	IQ	PANSS—P	PANSS—N	PANSS—G	PANSS Total
RMET total	-						
EQ total	0.756 **	-					
IQ	0.687 **	0.470 **	-				
PANSS—P	−0.177	−0.166	−0.130	-			
PANSS—N	−0.945 **	−0.681 **	−0.604 **	0.157	-		
PANSS—G	−0.871 **	−0.729 **	−0.468 **	0.220 *	0.863 **	-	
PANSS total	−0.924 **	−0.726 **	−0.529 **	0.304 **	0.941 **	0.934 **	-

RMET—Reading the Mind in the Eyes; EQ—Empathy Quotient; PANSS—Positive and Negative Symptom Scale; PANSS-P—Positive and Negative Symptom Scale—Positive symptoms; PANSS-N—Positive and Negative Symptom Scale—Negative symptoms; PANSS-G—Positive and Negative Symptom Scale—General symptoms; IQ—Intelligence quotient. * *p* < 0.05, ** *p* < 0.001.

**Table 6:** Hierarchical multiple regression analysis predicting RMET scores.

Predictor Block	B	SE	β	t	*p*	VIF	Model R^2^	Adjusted R^2^	ΔR^2^	F	ΔF
Model 1							0.490	0.475	0.490	33.61 **	33.61 **
(Constant)	−29.72	7.27	—	−4.09	<0.0001 **	—					
IQ (Intelligence)	0.53	0.07	0.69	7.55	<0.0001 **	1.14					
Sex	−0.58	1.56	−0.03	−0.37	0.709	1.14					
Model 2							0.715	0.703	0.225	57.67 **	54.46 **
(Constant)	−3.15	6.55	—	−0.48	0.632	—					
IQ (Intelligence)	0.31	0.06	0.40	5.07	<0.0001 **	1.50					
Sex	−1.34	1.18	−0.08	−1.14	0.258	1.15					
Metabolizer Status	−9.37	1.27	−0.55	−7.38	<0.0001 **	1.34					
Model 3							0.891	0.885	0.176	139.44 **	110.40 **
(Constant)	33.89	5.39	—	6.29	<0.0001 **	—					
IQ (Intelligence)	0.13	0.04	0.17	3.11	0.003 *	1.81					
Sex	1.05	0.77	0.06	1.36	0.177	1.26					
Metabolizer Status	−2.76	1.01	−0.16	−2.73	0.008 *	2.19					
PANSS (Negative)	−2.30	0.22	−0.72	−10.51	<0.0001 **	2.96					
Model 4							0.892	0.884	0.000	110.29 **	0.20
(Constant)	33.46	5.56	—	6.02	<0.0001 **	—					
IQ (Intelligence)	0.12	0.04	0.16	2.89	0.005 *	1.90					
Sex	0.51	0.75	0.03	0.68	0.502	1.17					
Metabolizer Status	−3.06	1.04	−0.18	−2.93	0.005 *	2.27					
PANSS (Negative)	−2.15	0.21	−0.70	−10.04	<0.0001 **	2.98					
Psychosocial Context	−0.03	0.83	−0.020	−0.04	0.969	1.25					

Durbin-Watson = 1.66. B—Unstandardized Coefficient; SE—Standard Error; β—Standardized Coefficient; t—T-statistic (test of individual predictor significance); VIF—Variance Inflation Factor; R^2^/ΔR^2^—Coefficient of determination (R-square)/R-square Change (explained variance); F/ΔF—F-statistic/F-ratio Change (test of overall model significance). * *p* < 0.05, ** *p* < 0.001.

**Table 7:** Hierarchical multiple regression analysis predicting total EQ scores.

Predictor Block	B	SE	β	t	*p*	VIF	Model R^2^	Adjusted R^2^	ΔR^2^	F	ΔF
Model 1							0.269	0.248	0.269	12.89 **	12.89 **
(Constant)	−7.79	8.03	—	−0.97	0.335	—					
IQ (Intelligence)	0.35	0.08	0.50	4.56	<0.0001 **	1.14					
Sex	−0.87	1.72	−0.06	−0.51	0.614	1.14					
Model 2							0.316	0.286	0.046	10.61 **	4.68 *
(Constant)	3.35	9.37	—	0.36	0.722	—					
IQ (Intelligence)	0.26	0.09	0.37	3.00	0.004 *	1.50					
Sex	−1.19	1.68	−0.08	−0.71	0.482	1.15					
Metabolizer Status	−3.93	1.82	−0.25	−2.16	0.034 *	1.34					
Model 3							0.475	0.444	0.159	15.34 **	20.62 **
(Constant)	35.84	10.93	—	3.28	0.002 *	—					
IQ (Intelligence)	0.10	0.08	0.15	1.23	0.223	1.81					
Sex	0.90	1.55	0.06	0.58	0.563	1.26					
Metabolizer Status	1.87	2.05	0.12	0.91	0.365	2.19					
PANSS (Negative)	−2.01	0.44	−0.69	−4.54	<0.0001 **	2.96					
Model 4							0.475	0.436	0.000	12.13 **	0.03
(Constant)	35.75	11.03	—	3.24	0.002 *	—					
IQ (Intelligence)	0.10	0.08	0.15	1.23	0.224	1.81					
Sex	0.92	1.57	0.06	0.59	0.559	1.27					
Metabolizer Status	1.96	2.13	0.12	0.92	0.363	2.33					
PANSS (Negative)	−2.01	0.45	−0.69	−4.49	<0.0001 **	2.98					
Psychosocial Context	−0.27	1.67	−0.02	−0.16	0.874	1.22					

Durbin-Watson = 1.81. B—Unstandardized Coefficient; SE—Standard Error; β—Standardized Coefficient; t—T-statistic (test of individual predictor significance); VIF—Variance Inflation Factor; R^2^/ΔR^2^—Coefficient of determination (R-square)/R-square Change (explained variance); F/ΔF—F-statistic/F-ratio Change (test of overall model significance). * *p* < 0.05, ** *p* < 0.001.

## Data Availability

Given the sensitive nature of the research, supporting data cannot be provided.
